# Supplementary material for: Colonization with multi-drug-resistant organisms negatively impacts survival in patients with non-small cell lung cancer
Source: PLoS One. 2020 Nov 25;15(11):e0242544. doi: 10.1371/journal.pone.0242544 (PMC7688109; doi:10.1371/journal.pone.0242544)
Supplement: S1 File — (DOCX) [file pone.0242544.s002.docx]

Colonization with multi-drug-resistant organisms negatively impacts survival in patients with non-small cell lung cancer

Jan A. Stratmann^1^*; Raphael Lacko^1^, Olivier Ballo^1^, Shabnam Shaid^1^, Wolfgang Gleiber^2^, Maria J.G.T. Vehreschild^3,7^, Thomas Wichelhaus^4,7,8^, Claudia Reinheimer^4,7,8^, Stephan Göttig^4,7^, Volkhard A. J. Kempf^4,7,8^ Peter Kleine^5^, Susanne Stera^6^, Christian Brandts^1,9^, Martin Sebastian^1^, Sebastian Koschade^1^

^1^ Department of Internal Medicine, Hematology/Oncology, Goethe University, Frankfurt, Frankfurt am Main, Germany

^2^ Department of Internal Medicine, Pneumology, Goethe University, Frankfurt, Frankfurt am Main, Germany

^3^ Department of Internal Medicine, Infectious Diseases, Goethe University, Frankfurt, Frankfurt am Main, Germany

^4^ Institute of Medical Microbiology and Infection Control, Goethe University, Frankfurt, Frankfurt am Main, Germany

^5^ Department of Cardiothoracic Surgery, Goethe University, Frankfurt, Frankfurt am Main, Germany

^6^ Department of Radiation Oncology, Goethe University, Frankfurt, Frankfurt am Main, Germany

^7^ University Center for Infectious Diseases, Goethe University, Frankfurt, Frankfurt am Main, Germany

^8^ University Center of Competence for Infection Control, State of Hesse, Germany

^9^ University Cancer Center Frankfurt (UCT), Goethe University, Frankfurt, Germany

*Corresponding author: Email: [jan.stratmann@kgu.de](mailto:jan.stratmann@kgu.de); (JS)

**Colonization with multi-drug-resistant organisms negatively impacts survival in patients with non-small cell lung cancer**

Supplement

**S1 Figure**: Flow diagram showing the process of inclusion of eligible patients into the analysis.

**S2 Table:**

Multivariate analysis of risk factors for MDRO colonization

| Risk Factor | Comparator |  | OR | 95% CI | P value |
| --- | --- | --- | --- | --- | --- |
| Diabetes mellitus | No diabetes mellitus |  | 1.60 | (0.66—2.55) | <0.001 |
| Heart disease | No heart disease |  | -0.21 | (-1.40—0.86) | .72 |
| Kidney disease | No kidney disease |  | 0.08 | (-1.05—1.11) | .88 |
| Liver disease | No liver disease |  | -0.46 | (-4.08—1.76) | .74 |
| Gender: male | Gender: female |  | 0.91 | (-0.14—2.20) | .12 |
| Age at diagnosis |  |  | 0.03 | (-0.01—0.08) | .25 |
| ECOG: 1 | ECOG: 0 |  | -0.06 | (-1.16—1.17) | .92 |
| ECOG: 2 | ECOG: 0 |  | 0.49 | (-0.92—1.93) | .50 |
| ECOG: 3 | ECOG: 0 |  | -14.47 |  | .99 |
| ECOG: 4 | ECOG: 0 |  | 3.07 | (-0.62—6.85) | .08 |
|  |  |  |  |  |  |

Logistical regression analysis. MDRO, multidrug resistance organism; OR, odds ratio; CI, confidence interval.

| **S3 Table:** | |  | | | | |  | | | | |  | | | | | | | |  |
| --- | --- | --- | --- | --- | --- | --- | --- | --- | --- | --- | --- | --- | --- | --- | --- | --- | --- | --- | --- | --- |
| Patient and disease characteristics, comparative off-target analysis | | | | | | | | | | | |  | | | | | | | |  |
|  | |  | | | | | Off-Target  population (n = 107) | | | | | Patients included  into analysis (n=295) | | | | | | | | P value* |
| Gender | | female | | | | | 46 (43%) | | | | | 110 (37%) | | | | | | | | .30 |
|  | | male | | | | | 61 (56%) | | | | | 185 (63%) | | | | | | | | .30 |
| Age at diagnosis, median (range), years | |  | | | | | 62 (32-83) | | | | | 67 (29-90) | | | | | | | | .001 |
| Smoking history | |  | | | | | 93 (87%) | | | | | 209 (71%) | | | | | | | | .0001 |
| ECOG performance score ≤ 2 (%) | |  | | | | | 96 (90%) | | | | | 281 (95%) | | | | | | | | .04 |
| Disease stage according to UICC 7th | | IA | | | | | 1 (1%) | | | | | 50 (17%) | | | | | | | | .00001 |
|  | | IB | | | | | 1 (1%) | | | | | 19 (6%) | | | | | | | |  |
|  | | IIA | | | | | 1 (1%) | | | | | 22 (7%) | | | | | | | |  |
|  | | IIB | | | | | 1 (1%) | | | | | 25 (8%) | | | | | | | |  |
|  | | IIIA | | | | | 15 (14%) | | | | | 61 (21%) | | | | | | | |  |
|  | | IIIB | | | | | 11 (10%) | | | | | 19 (6%) | | | | | | | |  |
|  | | IV | | | | | 77 (72%) | | | | | 99 (34%) | | | | | | | |  |
| Presence of brain metastases | |  | | | | | 42 (39%) | | | | | 48 (16%) | | | | | | | | .00001 |
| Histology | | Adeno NSCLC | | | | | 83 (78%) | | | | | 160 (54%) | | | | | | | |  |
|  | | SCNSCLC | | | | | 23 (21%) | | | | | 127 (43%) | | | | | | | |  |
|  | | other | | | | | 0 (0%) | | | | | 8 (3%) | | | | | | | |  |
| Mutations (pos. / neg.) | | ALK | | | | | 6 (6%) / 32 (30%) | | | | | 3 (3%) / 32 (11%) | | | | | | | |  |
|  | | BRAF | | | | | 0 (0%) /5 (5%) | | | | | 2 (1%) / 5 (2%) | | | | | | | |  |
|  | | EGFR | | | | | 21/70 (30%) | | | | | 15 (5%) / 33 (11%) | | | | | | | | .0001 |
|  | | KRAS | | | | | 9 (8%) / 21 (20%) | | | | | 15 (5%) / 14 (5%) | | | | | | | |  |
|  | | ROS1 | | | | | 4 (4%) / 15 (14%) | | | | | 4 (1%) / 12 (4%) | | | | | | | |  |
| Comorbidities | | Diabetes | | | | | 6 (6%) | | | | | 56 (19%) | | | | | | | | .004 |
|  | | HIV | | | | | 1 (1%) | | | | | 9 (3%) | | | | | | | | .23 |
|  | | Heart disease | | | | | 10 (9%) | | | | | 60 (20%) | | | | | | | | .013 |
|  | | Kidney disease | | | | | 19 (18%) | | | | | 52 (18%) | | | | | | | | .89 |
|  | | Liver disease | | | | | 0 (0%) | | | | | 9 (31%) | | | | | | | | .07 |
| Count data is shown unless indicated otherwise. *Differences between off-target population (missing MDRO screening) and analyzed patients were tested. Mann-Whitney U test was used to calculate P value for age. Except for EGFR, gene mutations were not tested due to missing data. SCNSCLC, squamous cell NSCLC | | | | | | | | | | | | | | | | | | | | |
| **S4 Table** | | | | | | | | | | | | | | | | | |  |  |  |
| Classifikation of MDRO according to resistance phenotype | | | | | | | | | | | | | | | | | |  |  |  |
| **Enterbacteriaceae spp.** | | | | | | | | | | | | | | | | | |  |  |  |
|  | **Spp.** | | **Phenotype** |  | **A** | **B** | | **C** | **D** | **E** | **F** | | **G** | **H** | **I** | **J** | |  |  |  |
| **1** | *E. coli* | | ESBL |  |  |  | |  | S |  | S | | S | S | S | S | |  |  |  |
| **2** | *E. coli* | | ESBL |  |  |  | |  |  |  | S | |  | S | NT | S | |  |  |  |
| **3** | *E. coli* | | ESBL |  |  |  | |  |  | S |  | | S | S | S | S | |  |  |  |
| **4** | *E. coli* | | ESBL |  |  |  | |  |  |  | S | | S | S | S | S | |  |  |  |
| **5** | *E. coli* | | ESBL |  |  |  | |  |  |  |  | | S | S | S | S | |  |  |  |
| **6** | *E. coli* | | ESBL |  |  |  | |  | S | S |  | |  | S |  | S | |  |  |  |
| **7** | *E. coli* | | ESBL |  |  |  | |  |  | S |  | | S | S | NT | S | |  |  |  |
| **8** | *E. coli* | | ESBL |  |  |  | |  |  |  | S | | S | S | S | S | |  |  |  |
| **9** | *E. coli* | | ESBL |  |  |  | |  |  |  |  | |  | S | S | S | |  |  |  |
| **10** | *K. pneumoniae* | | ESBL |  |  |  | |  |  |  |  | |  |  | S | S | |  |  |  |
| **11** | *E. coli* | | ESBL |  |  |  | |  |  |  | S | | S | S | S | S | |  |  |  |
| **12** | *K. pneumoniae* | | ESBL |  |  |  | |  |  |  |  | |  | S | NT | S | |  |  |  |
| **13** | *E. coli* | | ESBL |  |  |  | |  |  |  | S | |  | S | NT | S | |  |  |  |
| **14** | *E. coli* | | ESBL |  |  |  | |  |  | S | S | | S | S | S | S | |  |  |  |
| **15** | *E. coli* | | ESBL |  |  |  | |  |  |  |  | | S | S | S | S | |  |  |  |
| **16** | *E. coli* | | ESBL |  |  |  | |  |  | S | S | | S | S | S | S | |  |  |  |
| **17** | *E. coli* | | ESBL |  |  |  | |  |  | S | S | | S | S | S | S | |  |  |  |
| **18** | *E. coli* | | ESBL |  |  |  | |  |  | S |  | | S |  | S | S | |  |  |  |
| **19** | *K. pneumoniae* | | ESBL |  |  |  | |  |  |  |  | | S | S |  | S | |  |  |  |
| A, 1st/2nd generation cephalosporins; B, 3rd/4th generation cephalosporins; C, penicilline + lactamase inhibitor; D, antipseudomonale penicillins; E, fluorquinolones; F, folate pathway inhibitors; G, aminolykoside; H, glycylcycline, I, phosphonic acids; J, carbapenems | | | | | | | | | | | | | | | | | |  |  |  |
| **Enterococcus spp.** | | |  |  |  |  | |  |  |  |  | |  |  |  |  | |  |  |  |
|  |  | |  |  | **A** | **B** | | **C** | **D** | **E** | **F** | | **G** | **H** | **I** |  |  |  |  |  |
| **20** | *E. faecium* | | vanB |  |  |  | |  |  |  | S | | S | S | S |  |  |  |  |  |
| **21** | *E. faecium* | | vanA |  |  |  | |  |  | S | S | | S | S | S |  |  |  |  |  |
| **22** | *E. faecium* | | vanB |  |  |  | |  |  | S | S | | S | S | S |  |  |  |  |  |
| **23** | *E. faecium* | | vanB |  |  |  | |  |  | S |  | | S | S | S |  |  |  |  |  |
| A, carbapenems; B, penicillins; C, fluorquinolones; D, glycopeptides; E, aminoglycoside; F, tetracycline; G, glycylcyclines; H, lipopetides; I, oxazolidinones | | | | | | | | | | | | | | | |  | |  |  |  |
| ***S. aureus*** |  | |  |  |  |  | |  |  |  |  | |  |  |  |  | |  |  |  |
|  |  | |  |  | **A** | **B** | | **C** | **D** | **E** | **F** | | **I** | **J** | **K** | **L** | | **M** |  |  |
| **24** | *S. aureus* | | MRSA |  |  |  | |  | S | S | S | | S | S | S | S | | S |  |  |
| A, fluoroquinolones; B, lincosamides; C, macrolides; D, aminoglycosides; E, ansamycins; F, folate pathway inhibitors; G, glycopeptides; H, glycylcyclines; I, phosphonic acids; J, oxazolidinones; K, tetracyclines; L, anti-staphylococcal b-lactams; M, fucidanes | | | | | | | | | | | |  |  |  |  |  |  |  |  |  |
| MDRO, multidrug resistant organisms; spp., species; E. coli., Escherichia Coli; Klebsiella P., Klebsiella Pneumoniae; E. faecium; Enterococcus Faecium; ESBL, Extended Spectrum Beta Lactamase; VRE, Vancomycin Resistant Enterococcus; MRSA, Methicilline Resistant Staphylococcus Aureus; spp., species; NT, not tested; black box indicates phenotypical non-susceptibility to all tested agents in the antimicrobial category | | | | | | | | | | | |  |  |  |  |  |  |  |  |  |

**S5 Table**

Multivariate analysis of risk factors for event or death

| Risk Factor | Comparator |  | HR | 95% CI | P value |
| --- | --- | --- | --- | --- | --- |
| MDRO colonization | No colonization |  | 1.32 | (0.75—2.31) | .34 |
| Gender: male | Gender: female |  | 1.11 | (0.82—1.52) | .50 |
| Age at diagnosis |  |  | 1.00 | (—) | .48 |
| Extensive disease | Limited disease |  | 3.57 | (2.60—4.90) | <.001 |
| Histology: SCC | Histology: adenocarcinoma |  | 1.25 | (0.92—1.71) | .15 |
| Histology: others | Histology: adenocarcinoma |  | 0.86 | (0.35—2.12) | .74 |
| ECOG: 1 | ECOG: 0 |  | 1.14 | (0.80—1.63) | .47 |
| ECOG: 2 | ECOG: 0 |  | 1.79 | (1.16—2.76) | .01 |
| ECOG: 3 | ECOG: 0 |  | 3.77 | (1.15—12.36) | .03 |
| ECOG: 4 | ECOG: 0 |  | 0.79 | (0.17—3.68) | .76 |
| Diabetes mellitus | No diabetes mellitus |  | 1.46 | (1.00—2.11) | .05 |
|  |  |  |  |  |  |

Cox proportional hazard regression analysis. MDRO, multidrug resistant organisms; SCC, squamous cell carcinoma; HR, hazard ratio; CI, confidence interval.

**S6 Figure:** Total numbers of hospital admissions for patients with colonization by multidrug resistant organisms (MDRO) and without MDRO colonization due to (A) all causes; (B) cancer-related causes; (C) infections; (D) provision of supportive care; (E) causes related to other cancers; (F) miscellaneous causes. Boxplots indicate median (horizontal line), 25^th^ and 75^th^ percentiles (lower and upper hinges), range of data values at most 1.5 * inter-quartile range from the 25^th^ and 75^th^ percentiles (whiskers), and individual outliers. P values for differences between patient groups were calculated by negative binomial regression. A zero-inflated negative binomial regression model was chosen if superior fit was indicated by the Vuong non-nested test. ns, not significant.

**S7 Figure:** Total durations of hospitalization in days for patients with colonization by multidrug resistant organisms (MDRO) and without MDRO colonization due to (A) all causes; (B) cancer-related causes; (C) infections; (D) provision of supportive care; (E) causes related to other cancers; (F) miscellaneous causes. Boxplots indicate median (horizontal line), 25th and 75th percentiles (lower and upper hinges), range of data values at most 1.5 * inter-quartile range from the 25th and 75th percentiles (whiskers), and individual outliers. P values for differences between patient groups were calculated by negative binomial regression. A zero-inflated negative binomial regression model was chosen if superior fit was indicated by the Vuong non-nested test. ns, not significant.
