# Supplementary material for: Colonization with multi-drug-resistant organisms negatively impacts survival in patients with non-small cell lung cancer
Source: PLoS One. 2020 Nov 25;15(11):e0242544. doi: 10.1371/journal.pone.0242544 (PMC7688109; doi:10.1371/journal.pone.0242544)
Supplement: Attachment — Submitted filename: 201103 Response to Reviewers.docx [file pone.0242544.s004.docx]

Dear Prof. Heber, Dear Richard Hodge

thank you for the possibility to re-submit our manuscript after minor revision. We thank the referees for their valuable comments and have addressed all their concerns.

We hope the manuscript can now be accepted in *Plos One* and look forward to your response.

Sincerely,
Jan Stratmann, Sebastian Koschade

**Comments to the referees:**

Reviewer #1: The authors have considered all suggestions and corrections addressed by the reviewer. Comments have been added and additional references have been cited as suggested. Minor corrections have also been addressed. The most outstanding limitations were acknowledged. This makes the manuscript more acceptable from a readers point of view.
However, the author needs to correct one spelling mistake, that is 'thirdly' in line 326. Otherwise, the manuscript is acceptable.

***We thank the referee again for his time to review our revised manuscript and have applied the last suggested change.***

Reviewer #2: The authors have answered all queries. I have no further comments, The manuscript will be useful for the readers.

***We thank the reviewer for his valuable time.***
